# Integrated Mutation Profiling and Prognostic Genomic Signature in Pediatric Testicular and Ovarian Germ Cell Tumors

**DOI:** 10.1155/humu/3009537

**Published:** 2026-08-12

**Authors:** Xiaoqi Xuan, Yue Yang, Yongle Li, Jinlong Yang, Hang Wu, Yulong Gong, Guogen Li, Xiao Pu

**Affiliations:** ^1^ Department of Urological Surgery, Wuxi Children′s Hospital, Wuxi, Jiangsu, China

**Keywords:** chromosome 12p, event-free survival, *KRAS*, ovarian germ cell tumor, pediatric germ cell tumor, targeted sequencing, testicular germ cell tumor, *TP53*

## Abstract

**Background:**

Pediatric testicular and ovarian germ cell tumors generally have favorable outcomes, but a subset of patients experience relapse, progression, or persistent disease. Conventional risk assessment is based primarily on stage, histology, primary site, serum tumor markers, and treatment response; the incremental prognostic value of genomic profiling remains uncertain.

**Methods:**

This retrospective molecular clinicopathological study included 72 patients younger than 18 years with primary testicular or ovarian germ cell tumors diagnosed from January 2014 to December 2023. Pretreatment formalin‐fixed paraffin‐embedded (FFPE) tumor specimens underwent targeted next‐generation sequencing with a custom 89‐gene hybrid‐capture panel. One qualifying feature was counted for each pathogenic or likely pathogenic *TP53* mutation, chromosome 12p gain, *KRAS* amplification, and each additional pathogenic or likely pathogenic oncogenic alteration retained after annotation; tumors with two or more qualifying features were classified as signature‐positive. Event‐free survival (EFS) was the primary endpoint, and overall survival (OS) was secondary.

**Results:**

The cohort comprised 45 ovarian and 27 testicular tumors. Recurrent alterations included chromosome 12p gain; *KRAS*, *TP53*, *KIT*, and *NRAS* mutations; and *KRAS* amplification. Twenty tumors were signature‐positive, and 52 were signature‐negative. Events occurred in 35.0% versus 17.3%, and 3‐year EFS was 65.0% versus 88.5% (log‐rank *p* = 0.019). The stage‐adjusted hazard ratio for EFS was 2.74 (95% confidence interval, 1.01–7.43; *p* = 0.048). Three‐year OS was 85.0% versus 98.1% and did not differ significantly (*p* = 0.087).

**Conclusion:**

Targeted sequencing identified recurrent mutations and copy‐number alterations in pediatric gonadal germ cell tumors. The exploratory adverse genomic signature was associated with EFS but not OS. This hypothesis‐generating finding requires independent validation and does not establish mandatory testing, treatment escalation, or modification of first‐line therapy.

## 1. Introduction

Pediatric extracranial germ cell tumors arise from primordial germ cell lineages and occur at gonadal and extragonadal sites. Gonadal tumors in children and adolescents span mature and immature teratomas, yolk sac tumor, dysgerminoma or seminoma, embryonal carcinoma, choriocarcinoma, and mixed germ cell tumors. Diagnosis and management integrate primary site, histology, clinical stage, serum tumor markers, surgical findings, and treatment response [1, 2].

Clinical behavior varies with age, site, histological subtype, and disease extent. Cooperative‐group analyses have improved risk classification by combining these variables; however, they do not fully explain why some patients relapse despite apparently favorable features [2, 3]. Alpha‐fetoprotein (AFP) and beta‐human chorionic gonadotropin (*β*‐hCG) remain central to diagnosis, response assessment, and surveillance; dynamic AFP decline can add prognostic information beyond baseline clinicopathological features [4].

Treatment remains clinicopathology driven. Surgery is central for localized gonadal tumors, and platinum‐based chemotherapy is used for many malignant or advanced‐stage tumors. Because cure rates are high, risk‐adapted management must balance relapse prevention against fertility effects, acute toxicity, and long‐term survivorship [5–8]. A biomarker intended for clinical use must therefore add value beyond established risk factors and demonstrate that its use improves decisions or outcomes.

Pediatric germ cell tumors generally have a low point‐mutation burden but show recurrent alterations in developmental programs, *KIT*/RAS signaling, and copy number [9, 10]. In adults, integrated profiling of testicular germ cell tumors likewise found extensive aneuploidy with relatively few somatic mutations and significant *KIT*, *KRAS*, and *NRAS* mutations concentrated in tumors with seminoma components [11–13]. These parallels support molecular profiling, but age, site, histology, and developmental origin limit direct extrapolation from adult cohorts.

Chromosome 12p gain, including isochromosome 12p, is a characteristic feature of adult testicular germ cell tumors [14, 15]. Its frequency and prognostic interpretation are less certain in pediatric gonadal tumors, particularly when ovarian and testicular cases are pooled. Individual alterations are also infrequent, which makes single‐gene prognostic estimates unstable in modest pediatric cohorts.

The present study characterized recurrent alterations in FFPE specimens from pediatric testicular and ovarian germ cell tumors and evaluated an exploratory adverse genomic signature in relation to EFS and OS. The analysis was intended to generate a candidate risk hypothesis for independent validation, not to replace conventional staging or direct treatment.

## 2. Methods

### 2.1. Study Design and Case Identification

This retrospective molecular clinicopathological study was conducted at Wuxi Children′s Hospital. Consecutive patients with primary gonadal germ cell tumors diagnosed between January 2014 and December 2023 were identified from the institutional pathology archive, surgical records, and electronic medical records.

Eligible patients were younger than 18 years at diagnosis and had a pathologically confirmed primary testicular or ovarian germ cell tumor, available pretreatment FFPE tumor tissue, complete clinicopathological and follow‐up records, and a valid targeted‐sequencing result. Pretreatment tissue was obtained before systemic chemotherapy. Patients were excluded for an extragonadal primary tumor, recurrent disease without available primary pretreatment tissue, treatment before sampling, inadequate tissue, failed deoxyribonucleic acid (DNA) quality control, sequencing failure after repeat extraction, or missing follow‐up.

Targeted sequencing was performed retrospectively for research using eligible archived pretreatment specimens. Genetic testing was not mandatory in routine clinical care, and the genomic results were not used to determine conventional stage or risk group, surgery, chemotherapy, or surveillance.

Participants were stratified for analysis by primary site, age group, histological subtype, recorded Stages I–IV, and exploratory adverse genomic‐signature status. Age was grouped as ≤ 5, 6–12, and 13–17 years for descriptive analyses. Cox regression used age ≤ 12 versus 13–17 years and Stages I–II versus III–IV. These analytical strata were not used to assign treatment.

**Figure 1:**
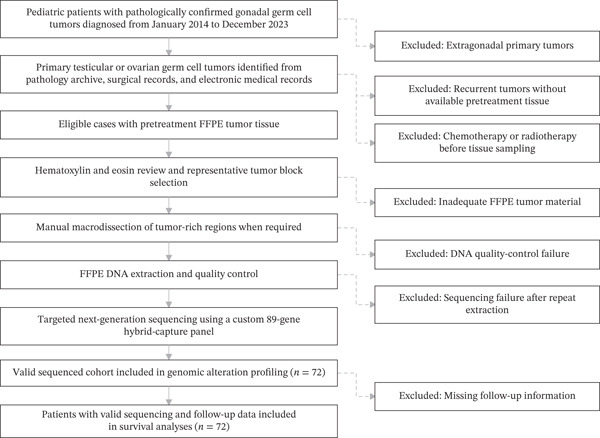
Study workflow and case selection. Pediatric patients with primary gonadal germ cell tumors diagnosed from January 2014 to December 2023 were screened. The valid sequenced cohort included 72 patients, all of whom had follow‐up data and were retained in the survival analyses. Exclusion criteria were an extragonadal primary tumor, unavailable pretreatment tissue, treatment before sampling, inadequate FFPE material, DNA quality‐control failure, sequencing failure, or missing follow‐up. FFPE, formalin‐fixed paraffin‐embedded; DNA, deoxyribonucleic acid.

All 72 patients with valid sequencing results and complete follow‐up, including the three patients with pure mature teratoma, were retained in the survival analyses. The study workflow and case‐selection process are shown in Figure 1.

### 2.2. Ethics Approval and Data Protection

The study was approved by the Ethics Committee of Wuxi Children′s Hospital (PWIU202377109). Written informed consent was waived because archived FFPE tissue and deidentified retrospective clinical data were used. Procedures complied with the Declaration of Helsinki and institutional requirements. Direct identifiers were removed from the analytic dataset, and the linkage file was stored separately with restricted access.

### 2.3. Clinicopathological Data Collection

Two trained reviewers independently extracted clinical and pathological information from electronic medical records, pathology reports, operative notes, chemotherapy records, imaging reports, laboratory records, and follow‐up documents. Discrepancies were resolved against the source record with a senior pediatric oncologist.

Collected variables and their operational definitions are provided by content domain in Table S1A,B. Age, tumor size, pretreatment AFP and *β*‐hCG, residual disease, treatment exposure, response, relapse, progression, EFS, and OS were defined before analysis.

Tumor size was the largest diameter on preoperative imaging; when unavailable, the largest gross pathological diameter was used. AFP was interpreted using age‐adjusted institutional reference ranges, and *β*‐hCG was classified using the institutional upper limit of normal.

### 2.4. Histopathological Review

Two pathologists experienced in pediatric solid tumors independently reviewed hematoxylin‐and‐eosin slides. Histological classification was based on the dominant malignant component, and mixed tumors were recorded when a component occupied at least 5% of tumor area. Disagreements were resolved by joint review.

One representative FFPE block containing at least 20% viable tumor was selected from each case. Necrotic, hemorrhagic, poorly fixed, cauterized, and decalcified regions were avoided. Manual macrodissection enriched tumor‐rich regions when required.

### 2.5. Treatment Variables

Treatment data were abstracted from operative records, chemotherapy orders, discharge summaries, and oncology follow‐up. Surgical procedure, residual disease, chemotherapy exposure, regimen, completed cycles, dose delays, dose reductions, and radiological response were recorded. Platinum‐based chemotherapy included documented bleomycin, etoposide, and cisplatin; carboplatin, etoposide, and bleomycin; cisplatin and etoposide with separately recorded bleomycin exposure; and other specified platinum combinations [6–8].

### 2.6. FFPE DNA Extraction and Quality Control

Six 8‐*μ*m unstained sections were cut from each selected block. The first and last sections were stained with hematoxylin and eosin to confirm tissue consistency. DNA was extracted with the QIAamp DNA FFPE Tissue Kit after xylene deparaffinization, ethanol washing, proteinase K digestion, and heat reversal of formalin crosslinks.

DNA concentration was measured using the Qubit dsDNA HS Assay, purity with the NanoDrop One spectrophotometer, and fragment distribution with the Agilent 2100 Bioanalyzer. Samples required at least 50 ng DNA, an A260/A280 ratio of 1.70–2.10, and a fragment profile compatible with hybrid capture. Failed samples were re‐extracted once from another section set or block.

### 2.7. Targeted Next‐Generation Sequencing

A custom 89‐gene hybrid‐capture panel targeted recurrent germ cell tumor alterations, signaling pathways, DNA repair genes, pediatric solid‐tumor genes, tumor suppressors, and selected chromosome 12p regions. Libraries were prepared with the KAPA HyperPlus kit and sequenced on an Illumina NextSeq 550 with paired‐end 150‐bp reads. Panel design, representative gene groups, bioinformatic resources, and quality‐control thresholds are summarized in Table S2.

A sample was accepted when mean deduplicated target depth was at least 300×, at least 90% of target bases had coverage of 100× or greater, the on‐target rate was at least 60%, and the duplicate‐read rate was below 70%. The run‐level Q30 metric denotes the percentage of bases with a Phred quality score of at least 30.

### 2.8. Bioinformatic Processing

Base‐call files were converted to FASTQ format with Illumina bcl2fastq v2.20. Quality was evaluated with FastQC v0.11.9; reads were trimmed with Trimmomatic v0.39, aligned to GRCh38 with BWA‐MEM v0.7.17, sorted and indexed with SAMtools v1.17, marked for duplicates with Picard MarkDuplicates v2.27.5, and recalibrated with Genome Analysis Toolkit v4.3.0.0.

Single‐nucleotide variants and small insertions/deletions were called with GATK Mutect2 in tumor‐only mode. Retained calls required depth ≥ 100×, alternate depth ≥ 10 reads, variant allele frequency ≥ 5%, mapping quality ≥ 30, and acceptable strand balance. Low‐complexity, low‐mappability, and recurrent artifact regions were excluded. Common variants were filtered using gnomAD, 1000 Genomes, and ExAC at a minor‐allele‐frequency threshold of 0.1%, except for variants classified as pathogenic or likely pathogenic in cancer databases.

Copy‐number alterations were inferred with CNVkit v0.9.10 using a panel‐of‐normals reference from FFPE samples processed with the same panel. Gene‐level gain, high‐level amplification, and loss were defined as estimated copy numbers ≥ 3.0, ≥ 5.0, and ≤ 1.5, respectively. Chromosome 12p gain required coordinated increases across at least three contiguous targeted regions and manual review of normalized coverage plots.

**Figure 2:**
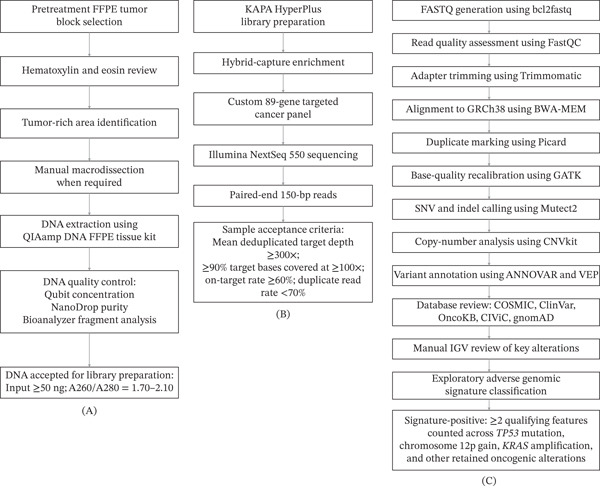
Targeted sequencing and bioinformatic workflow. (A) Pretreatment FFPE blocks underwent histological review, tumor enrichment, DNA extraction, and quality control. (B) Hybrid‐capture libraries were sequenced with paired‐end 150‐bp reads; acceptance required mean deduplicated depth ≥ 300×, ≥ 90% of targets at ≥ 100×, on‐target rate ≥ 60%, and duplicate rate < 70%. (C) Processing included FASTQ quality control, alignment to GRCh38, duplicate marking, base‐quality recalibration, single‐nucleotide variant, insertion/deletion, and copy‐number calling, annotation, database review, and manual review. Signature‐positive tumors had at least two qualifying features counted across *TP53* pathogenic or likely pathogenic mutation, chromosome 12p gain, *KRAS* amplification, and additional pathogenic or likely pathogenic oncogenic alterations. FFPE, formalin‐fixed paraffin‐embedded; DNA, deoxyribonucleic acid; BWA‐MEM, Burrows–Wheeler Aligner maximal exact matches; GATK, Genome Analysis Toolkit; VEP, Variant Effect Predictor; COSMIC, Catalogue of Somatic Mutations in Cancer; CIViC, Clinical Interpretation of Variants in Cancer; gnomAD, Genome Aggregation Database; IGV, Integrative Genomics Viewer.

Variants were annotated with ANNOVAR 2022Apr and Ensembl Variant Effect Predictor release 109 and reviewed against COSMIC, ClinVar, OncoKB, CIViC, dbSNP, and gnomAD. Key low‐frequency, hotspot, and signature‐defining calls were manually inspected in Integrative Genomics Viewer v2.16.2. The complete targeted‐sequencing and bioinformatic workflow is shown in Figure 2.

### 2.9. Variant Classification and Exploratory Genomic Grouping

Variants were classified with the four‐tier Association for Molecular Pathology/American Society of Clinical Oncology/College of American Pathologists framework. Tiers I–III variants were retained for descriptive genomic analyses; Tier IV variants were excluded [16].

An exploratory multihit adverse genomic signature was defined using the number of co‐occurring qualifying genomic features. One qualifying feature was counted for each pathogenic or likely pathogenic *TP53* mutation, chromosome 12p gain, *KRAS* amplification, and each additional pathogenic or likely pathogenic oncogenic alteration retained after annotation. Tumors harboring two or more qualifying features were classified as signature‐positive, whereas tumors with zero or one qualifying feature were classified as signature‐negative. Application of this rule classified 20 tumors as signature‐positive and 52 as signature‐negative.

### 2.10. Follow‐Up and Outcome Definitions

Follow‐up was obtained from oncology records, readmissions, imaging, laboratory results, multidisciplinary notes, and documented telephone contacts through December 31, 2024. Patients without an event were censored at the last confirmed contact; median follow‐up was estimated by reverse Kaplan–Meier analysis.

EFS was measured from first pathological diagnosis to relapse, progression, second‐line treatment for persistent disease, death from any cause, or last follow‐up. OS was measured from diagnosis to death from any cause or last follow‐up.

### 2.11. Statistical Analysis

Analyses were performed in R Version 4.3.2 and IBM SPSS Statistics Version 27.0. Continuous variables were summarized as mean ± standard deviation or median (interquartile range), as appropriate. Categorical variables were summarized as *n* (%). Site comparisons used Student′s *t* test or the Mann–Whitney *U* test for continuous variables and the chi‐square or two‐sided Fisher exact test for categorical variables; Fisher′s exact test was used for small expected counts.

For genomic frequency comparisons, the revised table reports the ovarian‐minus‐testicular prevalence difference in percentage points with Newcombe–Wilson 95% confidence intervals (CIs) and Cramér′s *V*, in addition to the two‐sided Fisher exact *p* value. These comparisons were exploratory and were not adjusted for multiplicity. The full statistical analysis framework is provided in Table S3.

Kaplan–Meier estimates and log‐rank tests were used for EFS and OS. Univariable Cox models examined clinicopathological and genomic variables. The main multivariable EFS model included stage and the exploratory adverse genomic signature because only 16 EFS events occurred. Hazard ratios (HRs) are presented with 95% CIs. All 72 patients were included in survival analyses, and complete‐case analysis was used without statistical imputation.

## 3. Results

### 3.1. Cohort Characteristics

The final sequenced cohort included 72 patients: 45 with ovarian tumors and 27 with testicular tumors. All had pretreatment FFPE tissue, valid targeted‐sequencing results, and follow‐up data.

Median age was 9.7 years. Patients with ovarian tumors were older than those with testicular tumors (10.8 vs. 6.4 years; *p* < 0.001), and age‐group distribution differed by site (*p* = 0.005). Stage distribution was similar between the groups (*p* = 0.956), with Stage I most common at both sites.

Ovarian tumors were larger than testicular tumors (median maximum diameter, 9.1 vs. 6.8 cm; descriptive median difference, 2.3 cm; *p* = 0.018). This difference may reflect site‐specific anatomy and detection patterns; the pooled 5‐cm threshold is therefore interpreted as exploratory rather than as a biologically equivalent cutoff for both sites.

Histological subtype differed by site (*p* = 0.002). Yolk sac tumor was most common overall; immature teratoma and dysgerminoma/seminoma occurred mainly in ovarian tumors, whereas mixed tumors and yolk sac tumors comprised a larger proportion of testicular cases. Pretreatment markers, residual disease, platinum exposure, event occurrence, and deaths were not significantly different by site (Table 1).

**Table 1:** Baseline clinicopathological characteristics of the sequenced cohort.

Characteristic	Total cohort	Ovarian tumors	Testicular tumors	*p*
Age at diagnosis, median (IQR), years	9.7 (6.1–12.5)	10.8 (8.2–13.4)	6.4 (2.8–9.6)	< 0.001
Age group, *n* (%)	—	—	—	0.005
≤ 5 years	14 (19.4)	4 (8.9)	10 (37.0)	
6–12 years	41 (56.9)	28 (62.2)	13 (48.1)	
13–17 years	17 (23.6)	13 (28.9)	4 (14.8)	
Maximum tumor diameter, median (IQR), cm	8.3 (7.0–10.2)	9.1 (7.6–11.0)	6.8 (5.4–8.7)	0.018
Stage, *n* (%)	—	—	—	0.956
I	32 (44.4)	21 (46.7)	11 (40.7)	
II	14 (19.4)	8 (17.8)	6 (22.2)	
III	18 (25.0)	11 (24.4)	7 (25.9)	
IV	8 (11.1)	5 (11.1)	3 (11.1)	
Histological subtype, *n* (%)	—	—	—	0.002
Yolk sac tumor	28 (38.9)	16 (35.6)	12 (44.4)	
Mixed germ cell tumor	16 (22.2)	8 (17.8)	8 (29.6)	
Immature teratoma	10 (13.9)	10 (22.2)	0 (0.0)	
Dysgerminoma/seminoma	10 (13.9)	9 (20.0)	1 (3.7)	
Embryonal carcinoma	5 (6.9)	2 (4.4)	3 (11.1)	
Mature teratoma	3 (4.2)	0 (0.0)	3 (11.1)	
Pretreatment AFP, median (IQR), ng/mL	246.8 (42.5–1164.0)	180.5 (35.2–980.3)	420.3 (88.6–1486.5)	0.112
Pretreatment *β*‐hCG, median (IQR), mIU/mL	2.7 (1.2–8.4)	2.5 (1.1–7.9)	3.1 (1.4–9.2)	0.674
Pretreatment LDH, median (IQR), U/L	278 (212–386)	265 (208–374)	295 (224–402)	0.421
Residual disease after surgery, *n* (%)	12 (16.7)	8 (17.8)	4 (14.8)	1.000
Platinum‐based chemotherapy, *n* (%)	56 (77.8)	33 (73.3)	23 (85.2)	0.380
Event during follow‐up, *n* (%)	16 (22.2)	10 (22.2)	6 (22.2)	1.000
Death during follow‐up, *n* (%)	4 (5.6)	3 (6.7)	1 (3.7)	1.000

*Note:* Values are *n* (%) unless otherwise stated.

Abbreviations: *β*‐hCG, beta‐human chorionic gonadotropin; AFP, alpha‐fetoprotein; IQR, interquartile range; LDH, lactate dehydrogenase.

### 3.2. Sequencing Profile and Recurrent Genomic Alterations

All 72 tumors passed the prespecified sequencing quality‐control thresholds.

**Figure 3:**
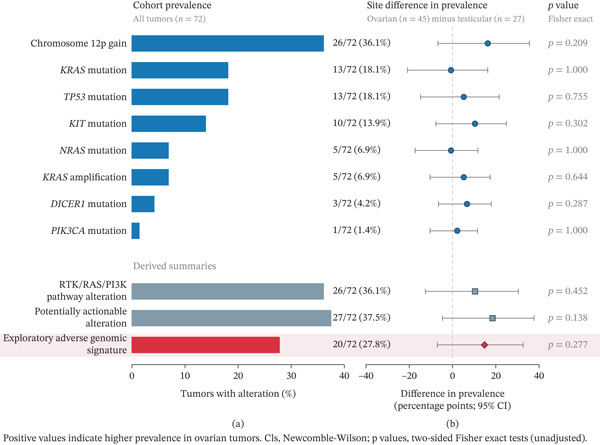
Aggregate genomic alteration frequencies and site differences. (a) Frequencies in the 72‐patient cohort. (b) Ovarian‐minus‐testicular prevalence differences with Newcombe–Wilson 95% CIs; *p* values are from two‐sided Fisher exact tests. Positive differences indicate a higher prevalence in ovarian tumors. The exploratory adverse genomic signature required at least two qualifying features. RTK, receptor tyrosine kinase; PI3K, phosphatidylinositol 3‐kinase; CI, confidence interval.

Recurrent alterations included chromosome 12p gain in 26 tumors; *KRAS* and *TP53* mutations in 13 each; *KIT* mutation in 10; *NRAS* mutation and *KRAS* amplification in 5 each; *DICER1* mutation in 3; and *PIK3CA* mutation in 1. Aggregate alteration frequencies and ovarian–testicular prevalence differences are shown in Figure 3.

At the pathway or derived‐feature level, RTK/RAS/PI3K alterations occurred in 26 tumors, potentially actionable alterations in 27, and the exploratory adverse genomic signature in 20. None of the displayed ovarian‐versus‐testicular comparisons reached statistical significance (Table 2).

**Table 2:** Genomic alterations detected by targeted sequencing, with site‐comparison effect sizes.

Genomic alteration	Total *n* = 72	Ovarian *n* = 45	Testicular *n* = 27	Difference, pp (95% CI)	Cramér′s *V*	*p*
Chromosome 12p gain	26 (36.1)	19 (42.2)	7 (25.9)	16.3 (−6.7 to 35.6)	0.164	0.209
KRAS mutation	13 (18.1)	8 (17.8)	5 (18.5)	−0.7 (−20.8 to 16.3)	0.009	1.000
TP53 mutation	13 (18.1)	9 (20.0)	4 (14.8)	5.2 (−14.7 to 21.6)	0.065	0.755
KIT mutation	10 (13.9)	8 (17.8)	2 (7.4)	10.4 (−7.7 to 24.9)	0.145	0.302
NRAS mutation	5 (6.9)	3 (6.7)	2 (7.4)	−0.7 (−17.3 to 11.7)	0.014	1.000
KRAS amplification	5 (6.9)	4 (8.9)	1 (3.7)	5.2 (−10.4 to 17.4)	0.099	0.644
DICER1 mutation	3 (4.2)	3 (6.7)	0 (0.0)	6.7 (−6.5 to 17.9)	0.162	0.287
PIK3CA mutation	1 (1.4)	1 (2.2)	0 (0.0)	2.2 (−10.4 to 11.6)	0.092	1.000
RTK/RAS/PI3K pathway alteration	26 (36.1)	18 (40.0)	8 (29.6)	10.4 (−12.5 to 30.4)	0.105	0.452
Potentially actionable alteration	27 (37.5)	20 (44.4)	7 (25.9)	18.5 (−4.6 to 37.7)	0.185	0.138
Exploratory adverse genomic signature	20 (27.8)	15 (33.3)	5 (18.5)	14.8 (−7.0 to 32.7)	0.160	0.277

*Note:* Values are *n* (%). Differences are ovarian minus testicular proportions in percentage points; 95% CIs use the Newcombe–Wilson method. *p* values are from two‐sided Fisher exact tests. Comparisons are exploratory and unadjusted for multiplicity. The RTK/RAS/PI3K *p* value is 0.452 when recalculated from the displayed counts. Potentially actionable alterations included *KIT*, *KRAS*, *NRAS*, or *PIK3CA* mutation or *KRAS* amplification. The exploratory adverse genomic signature required at least two qualifying features as defined in methods.

Abbreviations: CI, confidence interval; PI3K, phosphatidylinositol 3‐kinase; pp, percentage points; RTK, receptor tyrosine kinase.

### 3.3. Exploratory Adverse Genomic Signature and Survival Outcomes

Twenty tumors were signature‐positive, and 52 were signature‐negative. Events occurred in 7 of 20 (35.0%) signature‐positive tumors and 9 of 52 (17.3%) signature‐negative tumors. Median follow‐up for EFS was 36.2 months, with 16 events. Three‐year EFS was 65.0% versus 88.5% (log‐rank *p* = 0.019).

**Figure 4:**
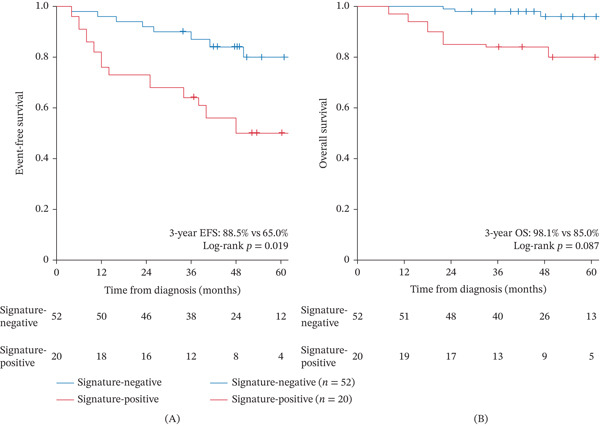
Survival outcomes according to exploratory adverse genomic‐signature status. (A) Kaplan–Meier estimates of EFS for signature‐negative (*n* = 52) and signature‐positive (*n* = 20) tumors; 3‐year EFS was 88.5% versus 65.0% (log‐rank *p* = 0.019). (B) Kaplan–Meier estimates of OS; 3‐year OS was 98.1% versus 85.0% (log‐rank *p* = 0.087; four deaths). Signature‐positive tumors had at least two qualifying genomic features. Tick marks denote censored observations; numbers below the plots show patients at risk. EFS, event‐free survival; OS, overall survival.

Four deaths occurred. Three‐year OS was 85.0% in the signature‐positive group and 98.1% in the signature‐negative group; the log‐rank comparison was not statistically significant (*p* = 0.087). Kaplan–Meier estimates for EFS and OS are shown in Figure 4.

### 3.4. Cox Regression for Event‐Free Survival

The exploratory adverse genomic signature was associated with poorer EFS in univariable Cox regression (HR 3.28, 95% CI 1.18–9.11; *p* = 0.023). Stages III–IV disease, residual disease, metastasis, chromosome 12p gain, *TP53* mutation, and *KRAS* amplification had HRs above 1.0, but their CIs included 1.0.

**Figure 5:**
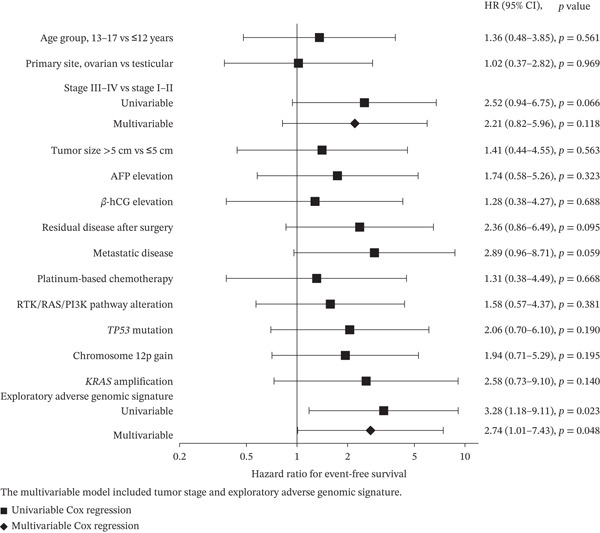
Cox regression for EFS. Squares indicate univariable HRs and diamonds indicate multivariable HRs; horizontal lines are 95% CIs and the vertical line marks HR = 1.0. The multivariable model included stage and the exploratory adverse genomic signature. Reference groups are shown in each comparison. The analysis included all 72 patients and 16 EFS events. HR, hazard ratio; CI, confidence interval; EFS, event‐free survival; AFP, alpha‐fetoprotein; *β*‐hCG, beta‐human chorionic gonadotropin; RTK, receptor tyrosine kinase; PI3K, phosphatidylinositol 3‐kinase.

After adjustment for stage, the signature HR was 2.74 (95% CI 1.01–7.43; *p* = 0.048). The wide CI, borderline lower bound, limited event count, and absence of external validation indicate an exploratory prognostic association rather than established clinical utility. No treatment interaction was tested, and signature status did not direct treatment. The corresponding univariable and multivariable HR estimates are shown in Figure 5 and reported in Table 3.

**Table 3:** Cox regression analysis for event‐free survival.

Variable	Univariable HR (95% CI)	*p*	Multivariable HR (95% CI)	*p*
Age group, 13–17 versus ≤ 12 years	1.36 (0.48–3.85)	0.561	—	—
Primary site, ovarian versus testicular	1.02 (0.37–2.82)	0.969	—	—
Stages III–IV versus Stages I–II	2.52 (0.94–6.75)	0.066	2.21 (0.82–5.96)	0.118
Tumor size > 5 versus ≤ 5 cm	1.41 (0.44–4.55)	0.563	—	—
AFP elevation	1.74 (0.58–5.26)	0.323	—	—
*β*‐hCG elevation	1.28 (0.38–4.27)	0.688	—	—
Residual disease after surgery	2.36 (0.86–6.49)	0.095	—	—
Metastatic disease	2.89 (0.96–8.71)	0.059	—	—
Platinum‐based chemotherapy	1.31 (0.38–4.49)	0.668	—	—
RTK/RAS/PI3K pathway alteration	1.58 (0.57–4.37)	0.381	—	—
TP53 mutation	2.06 (0.70–6.10)	0.190	—	—
Chromosome 12p gain	1.94 (0.71–5.29)	0.195	—	—
KRAS amplification	2.58 (0.73–9.10)	0.140	—	—
Exploratory adverse genomic signature	3.28 (1.18–9.11)	0.023	2.74 (1.01–7.43)	0.048

*Note:* The multivariable model included stage and the exploratory adverse genomic signature. All 72 patients were included; 16 EFS events informed the model.

Abbreviations: *β*‐hCG, beta‐human chorionic gonadotropin; AFP, alpha‐fetoprotein; CI, confidence interval; HR, hazard ratio; RTK, receptor tyrosine kinase; PI3K, phosphatidylinositol 3‐kinase.

## 4. Discussion

This study characterized clinicopathological features and targeted genomic alterations in 72 pediatric gonadal germ cell tumors. The principal findings were recurrent chromosome 12p gain and *KIT*/RAS‐related alterations, clear age, size, and histological differences between ovarian and testicular tumors, and an exploratory association between a multihit adverse genomic signature and EFS.

The genomic pattern broadly resembles prior pediatric studies[17–19]. Kubota et al. [9] identified age‐ and histology‐related molecular heterogeneity in 51 pediatric germ cell tumors using genomic, transcriptomic, and methylation assays. A recent matched‐normal whole‐exome study of 16 pediatric tumors likewise reported low mutational burden, *KIT*/RAS and PI3K/AKT‐pathway alterations, and copy‐number changes that included chromosome 12 [10]. Adult testicular germ cell tumors also show high aneuploidy with relatively few somatic mutations, but significant *KIT*, *KRAS*, and *NRAS* mutations are concentrated in seminoma‐containing tumors and occur within distinct epigenomic subtypes [13, 20]. The present pooled pediatric gonadal cohort cannot establish that adult molecular associations apply uniformly across age, site, and histology.

Chromosome 12p gain was the most frequent alteration, consistent with its established role in germ cell tumor development [14, 15]. However, a recurrent feature is not necessarily a prognostic biomarker. The individual HRs for 12p gain, *TP53* mutation, and *KRAS* amplification were imprecise and nonsignificant. *TP53*‐associated adverse outcomes have been reported in adult primary mediastinal nonseminomatous tumors [21], but that disease context differs from pediatric gonadal tumors. Combining infrequent alterations may improve numerical stability, yet a signature requires a reproducible locked definition, calibration, discrimination, and external validation before clinical use.

The EFS and OS findings are not clinically contradictory. EFS accrued 16 first events, including relapse, progression, second‐line treatment for persistent disease, and death, whereas OS accrued only four deaths. EFS therefore contained more information about early disease‐control failure. Salvage therapy after recurrence could also preserve OS, although this mechanism was not tested. The signature may identify earlier disease‐control failure, but the study does not establish an effect on mortality and does not justify treatment escalation.

Targeted sequencing was a retrospective research procedure and was not mandatory for routine management. The study did not evaluate whether genomic testing improved risk classification beyond established variables, changed treatment, or predicted benefit from a specific therapy. Similarly, ovarian tumors were 2.3 cm larger in median diameter than testicular tumors, but site‐specific anatomy and detection patterns make a pooled 5‐cm cutoff difficult to interpret. The tumor‐size HR was therefore exploratory, and larger cohorts are needed for continuous or site‐standardized size modeling [22].

Recent pediatric and ovarian germ cell tumor studies demonstrate histology‐dependent immune microenvironments, including immune‐active dysgerminoma and more immune‐desert or stromal phenotypes in other subtypes [23–25]. These observations support genomic–immune integration as a future research question. Because the present sequencing cohort did not include a prespecified quantitative immune analysis, immune relationships are presented only as a hypothesis‐generating framework in Figure S1 and are not interpreted as empirical associations.

Several limitations constrain inference. The study was retrospective and single‐center, and only 16 EFS events limited multivariable modeling. Tumor‐only sequencing cannot fully exclude rare germline variants. Ovarian and testicular tumors were pooled despite age, size, and histological differences, and site‐specific event counts were inadequate for stable models. The adverse genomic signature was exploratory, and its performance, reproducibility, and incremental value beyond established clinical factors require independent validation.

Future work should validate the locked signature rule in an independent multicenter pediatric cohort, report calibration and discrimination in addition to association, prespecify site and histology analyses, and test whether the biomarker adds information beyond stage and tumor‐marker kinetics. Treatment modification should be considered only after prospective evidence demonstrates clinical benefit.

## 5. Conclusion

Targeted sequencing identified recurrent mutations and copy‐number alterations in pediatric testicular and ovarian germ cell tumors. The exploratory adverse genomic signature was associated with EFS but not OS. This finding is hypothesis‐generating and requires independent multicenter validation before the signature can be considered for clinical risk classification. The present study does not establish mandatory testing, incremental clinical utility, or a basis for changing treatment.

## Author Contributions

Xiaoqi Xuan and Yue Yang contributed equally and share first authorship.

## Funding

No funding was received for this manuscript.

## Ethics Statement

The study was approved by the Ethics Committee of Wuxi Children′s Hospital (PWIU202377109). Written informed consent was waived for this retrospective use of archived FFPE tissue and deidentified clinical data. Procedures complied with the Declaration of Helsinki and institutional requirements.

## Conflicts of Interest

The authors declare no conflicts of interest.

## Supporting Information

Additional supporting information can be found online in the Supporting Information section.

## Supporting information


**Supporting Information 1** The supporting material file contains Table S1: (A) Clinical and treatment variable definitions and (B) genomic, follow‐up, and outcome variable definitions; Table S2: Representative targeted‐panel content, quality‐control thresholds, software, and databases; and Table S3: Statistical analysis framework.

**Supporting Information 2** Figure S1: Hypothesis‐generating genomic–microenvironment framework.

## Data Availability

De‐identified clinical and sequencing data are not publicly available because of ethical and institutional restrictions concerning pediatric participants. Data may be available from the corresponding author on reasonable request and with institutional permission.
