# Supplementary material for: Integrated Mutation Profiling and Prognostic Genomic Signature in Pediatric Testicular and Ovarian Germ Cell Tumors
Source: Hum Mutat. 2026 Aug 12;2026:3009537. doi: 10.1155/humu/3009537 (PMC13462903; doi:10.1155/humu/3009537)
Supplement: Supplementary file 1 [file HUMU-2026-3009537-s002.docx]

**Supplementary Tables and Figure**

Integrated Mutation Profiling and Prognostic Genomic Signature in Pediatric Testicular and Ovarian Germ Cell Tumors

**Supplementary Table S1A. Clinical and treatment variable definitions**

| **Category** | **Variable** | **Description** | **Data Source** | **Values** |
| --- | --- | --- | --- | --- |
| Histology | Germ cell tumor components | One or more germ cell tumor components, with each recorded component occupying at least 5% of total tumor area | Pathology report; slide review | Present; absent |
| Histology | Malignant component | Presence of yolk sac tumor, dysgerminoma/seminoma, embryonal carcinoma, choriocarcinoma, or malignant mixed germ cell tumor component | Pathology report; slide review | Present; absent |
| Tumor stage | Stage | Stage assigned using operative findings, imaging findings, pathology report, and oncology record | Imaging report; operative note; pathology report; oncology record | I; II; III; IV |
| Disease extent | Lymph node involvement | Regional lymph node disease documented by imaging, pathology, or oncology assessment | Imaging report; pathology report; oncology note | Present; absent |
| Disease extent | Distant metastasis | Tumor spread outside the primary gonadal site and regional lymph nodes | Imaging report; oncology note | Present; absent |
| Pretreatment marker | AFP | Serum alpha-fetoprotein measured within 14 days before surgery or diagnostic biopsy | Laboratory information system | Continuous value; elevated vs normal according to age-adjusted institutional reference range |
| Pretreatment marker | β-hCG | Serum beta-human chorionic gonadotropin measured within 14 days before surgery or diagnostic biopsy | Laboratory information system | Continuous value; elevated vs normal according to institutional reference range |
| Pretreatment marker | LDH | Serum lactate dehydrogenase measured within 14 days before surgery or diagnostic biopsy | Laboratory information system | Continuous value; elevated vs normal according to institutional reference range |
| Surgery | Initial surgical procedure | First tumor-directed surgical procedure performed before systemic treatment | Operative note | Orchiectomy; ovarian cystectomy; oophorectomy; salpingo-oophorectomy; tumor excision; biopsy only |
| Surgery | Fertility-sparing surgery | Procedure preserving the contralateral gonad and reproductive tract where anatomically applicable | Operative note | Yes; no |
| Surgery | Residual disease after surgery | Macroscopic residual tumor documented in operative note or postoperative imaging after initial surgery | Operative note; postoperative imaging report | Present; absent |
| Treatment | Chemotherapy exposure | Receipt of tumor-directed systemic chemotherapy after pathological diagnosis | Chemotherapy administration record; discharge summary | No chemotherapy; platinum-based chemotherapy; other chemotherapy |
| Treatment | Treatment pathway | Combination of surgery and systemic therapy received after diagnosis | Operative note; chemotherapy record | Surgery alone; surgery plus platinum-based chemotherapy; biopsy plus platinum-based chemotherapy; no tumor-directed treatment |
| Treatment | Chemotherapy regimen | Drug combination documented in completed chemotherapy administration records | Chemotherapy administration record | BEP; JEB; PEb; other platinum-based regimen |
| Treatment | Number of chemotherapy cycles | Total completed cycles recorded in medication administration records | Chemotherapy administration record | 0; 1–2; 3–4; >4 cycles |
| Treatment | Dose delay | Chemotherapy cycle interval extended by more than 7 days for documented clinical reasons | Chemotherapy order; oncology note | Present; absent |
| Treatment | Dose reduction | Explicit reduction in chemotherapy dose documented because of toxicity, organ dysfunction, infection, or poor tolerance | Chemotherapy order; oncology note | Present; absent |
| Response | Radiological response | Tumor response documented by post-treatment imaging and oncology assessment | Imaging report; oncology note | Complete response; partial response; stable disease; progressive disease |

AFP = alpha-fetoprotein; β-hCG = beta-human chorionic gonadotropin; LDH = lactate dehydrogenase; BEP = bleomycin, etoposide, and cisplatin; JEB = carboplatin, etoposide, and bleomycin; PEb = cisplatin and etoposide with separately recorded bleomycin exposure.

**Supplementary Table S1B. Genomic, follow-up, and outcome variable definitions**

| **Category** | **Variable** | **Description** | **Data Source** | **Values** |
| --- | --- | --- | --- | --- |
| Genomic alteration | Recurrent genomic alteration | Tier I–III sequence variant or copy-number alteration retained after filtering and annotation | Sequencing report; bioinformatic output | Gene-level alteration; pathway-level alteration |
| Genomic alteration | RTK/RAS/PI3K pathway alteration | Presence of retained alteration in KIT, KRAS, NRAS, HRAS, BRAF, PIK3CA, PTEN, or AKT1 | Sequencing report; bioinformatic output | Present; absent |
| Genomic alteration | TP53 mutation | Pathogenic or likely pathogenic TP53 sequence variant retained after filtering | Sequencing report; bioinformatic output; manual IGV review | Present; absent |
| Genomic alteration | Chromosome 12p gain | Coordinated copy-number increase involving at least three contiguous 12p target regions after normalization | CNVkit output; normalized coverage plot | Present; absent |
| Genomic alteration | KRAS amplification | Estimated KRAS copy number of at least 5.0 after normalized copy-number analysis | CNVkit output; normalized coverage plot | Present; absent |
| Genomic grouping | Exploratory adverse genomic signature | One qualifying feature for each TP53 pathogenic or likely pathogenic mutation, chromosome 12p gain, KRAS amplification, and each additional pathogenic or likely pathogenic oncogenic alteration; signature-positive at two or more total qualifying features | Sequencing report; bioinformatic output | Signature-positive; signature-negative |
| Follow-up | Relapse | Recurrent disease after complete response or no evidence of disease, confirmed by imaging, pathology, tumor-marker progression, or oncology documentation | Imaging report; pathology report; laboratory record; oncology note | Present; absent |
| Follow-up | Progression | Enlargement of residual measurable disease, new lesion, or persistent marker increase interpreted as progressive disease by the treating team | Imaging report; laboratory record; oncology note | Present; absent |
| Follow-up | Second-line treatment for persistent disease | Initiation of additional tumor-directed treatment because of persistent disease after first-line treatment | Oncology note; chemotherapy record | Present; absent |
| Survival endpoint | Event-free survival | Time from first pathological diagnosis to relapse, progression, second-line treatment for persistent disease, death from any cause, or last follow-up | Pathology report; follow-up record | Months |
| Survival endpoint | Overall survival | Time from first pathological diagnosis to death from any cause or last follow-up | Follow-up record; hospital record | Months |
| Censoring | Last follow-up date | Most recent confirmed clinical contact before the follow-up cutoff date of December 31, 2024 | Outpatient record; inpatient record; telephone follow-up documentation | Date |
| Censoring | Event-free censoring | No relapse, progression, second-line treatment, or death before last follow-up | Follow-up record | Censored |
| Censoring | Overall survival censoring | Alive at last follow-up | Follow-up record | Censored |

RTK = receptor tyrosine kinase; PI3K = phosphatidylinositol 3-kinase; IGV = Integrative Genomics Viewer; EFS = event-free survival; OS = overall survival.

Supplementary Table S2. Representative targeted-panel content, quality-control thresholds, software, and databases

| **Category** | **Subcategory** | **Details** | **Standard/Criteria** |
| --- | --- | --- | --- |
| Representative panel genes | Included genes | BRAF, TP53, PIK3CA, PTEN, AKT1, CTNNB1, DICER1, TNFRSF8 | Representative genes shown |
| RTK/RAS/PI3K pathway genes | Included genes | KIT, KRAS, NRAS, HRAS, BRAF, PIK3CA, PTEN, AKT1 | Grouped as RTK/RAS/PI3K pathway alterations |
| DNA repair genes | Included genes | ATM, ATR, BRCA1, BRCA2, PALB2, CHEK2, POLE, POLD1 | Representative genes shown |
| Pediatric solid tumor genes | Included genes | ALK, MET, RET, ROS1, NTRK1, NTRK2, NTRK3, SMARCA4, SMARCB1, ARID1A | Representative genes shown |
| Cell-cycle and tumor suppressor genes | Included genes | CDKN2A, RB1, MDM2, CCND2 | Representative genes shown |
| Copy-number loci | Chromosome 12p targets | Selected target regions across chromosome 12p | Used for chromosome 12p gain assessment |
| Run-level QC | Sequencing run quality | Total yield, Q30 score, cluster density, and internal control metrics | Run accepted only when manufacturer’s run-quality requirements were met |
| Sample-level QC | Mean target depth | Mean deduplicated sequencing depth across target regions | ≥300× |
| Sample-level QC | Target-base coverage | Proportion of target bases covered at sufficient depth | ≥90% of target bases covered at ≥100× |
| Sample-level QC | On-target rate | Proportion of reads mapped to targeted regions | ≥60% |
| Sample-level QC | Duplicate read rate | Proportion of duplicate reads after alignment | <70% |
| FASTQ generation | Conversion software | Illumina bcl2fastq v2.20 | FASTQ files generated from raw base-call files |
| FASTQ QC | Quality assessment | FastQC v0.11.9 | Per-sample FASTQ quality report generated |
| Read trimming | Trimming software | Trimmomatic v0.39 | Adapter sequences and low-quality bases removed |
| Alignment | Reference genome and aligner | GRCh38; BWA-MEM v0.7.17 | Clean reads aligned to GRCh38 |
| Alignment processing | Sorting and indexing | SAMtools v1.17 | Sorted and indexed BAM files generated |
| Duplicate marking | Duplicate processing tool | Picard MarkDuplicates v2.27.5 | Duplicate reads marked before variant calling |
| Base recalibration | Recalibration tool | GATK v4.3.0.0 | Base-quality score recalibration completed |
| SNV/indel calling | Variant caller | GATK Mutect2 v4.3.0.0 in tumor-only mode | Candidate SNVs and indels generated |
| SNV/indel filtering | Total read depth | Read depth at variant locus | ≥100× |
| SNV/indel filtering | Alternate allele depth | Number of reads supporting alternate allele | ≥10 reads |
| SNV/indel filtering | Variant allele frequency | Proportion of reads supporting alternate allele | ≥5% |
| SNV/indel filtering | Mapping quality | Mapping-quality threshold | ≥30 |
| Population filtering | Germline/common variant filtering | gnomAD, 1000 Genomes, and ExAC | MAF <0.1%, unless listed as pathogenic or likely pathogenic in a cancer database |
| Copy-number analysis | CNV caller | CNVkit v0.9.10 | Panel-of-normals reference constructed from FFPE samples processed with the same capture workflow |
| Copy-number gain | Gene-level gain | Estimated copy number from normalized target depth | Copy number ≥3.0 |
| High-level amplification | Gene-level amplification | Estimated high-level copy-number increase | Copy number ≥5.0 |
| Copy-number loss | Gene-level loss | Estimated copy-number decrease | Copy number ≤1.5 |
| Chromosome 12p gain | Calling rule | Coordinated copy-number increase across chromosome 12p targets | ≥3 contiguous 12p target regions with increased copy number |
| Variant annotation | Annotation tools | ANNOVAR 2022Apr; Ensembl Variant Effect Predictor release 109 | Annotated variant table generated |
| Database review | Cancer and population databases | COSMIC, ClinVar, OncoKB, CIViC, dbSNP, and gnomAD | Used for interpretation and filtering |
| Manual review | Visualization tool | Integrative Genomics Viewer v2.16.2 | Required for TP53 variants, hotspot variants, KRAS amplification, chromosome 12p gain, and signature-defining alterations |
| Variant classification | Classification framework | AMP/ASCO/CAP four-tier somatic variant interpretation framework | Tier I–III retained; Tier IV excluded |
| Genomic signature | Signature-positive definition | One qualifying feature for each TP53 pathogenic or likely pathogenic mutation, chromosome 12p gain, KRAS amplification, and each additional pathogenic or likely pathogenic oncogenic alteration; signature-positive at ≥2 total qualifying features | Signature-positive if ≥2 qualifying features were present |

**Supplementary Table S3. Statistical analysis framework**

| **Analysis Type** | **Description** | **Method/Definition** | **Representation** |
| --- | --- | --- | --- |
| Continuous variables | Normally distributed variables | Summarized as mean ± standard deviation | Mean ± SD |
| Continuous variables | Non-normally distributed variables | Summarized as median and interquartile range | Median (IQR) |
| Categorical variables | Descriptive summary | Summarized as frequency and percentage | n (%) |
| Site comparison | Ovarian versus testicular tumors | Student’s t test, Mann–Whitney U test, chi-square test, or Fisher’s exact test according to variable type and distribution | Test statistic and P value |
| Small expected cell counts | Categorical comparison rule | Fisher’s exact test used when any expected cell count was below 5 | P value |
| Genomic frequency analysis | Gene-level alteration frequency | Number of cases with retained Tier I–III alteration in each gene divided by number of successfully sequenced cases | n (%) |
| Genomic frequency analysis | Pathway-level alteration frequency | Presence of at least one retained alteration in a predefined pathway gene set | n (%) |
| RTK/RAS/PI3K pathway grouping | Pathway definition | Alteration in KIT, KRAS, NRAS, HRAS, BRAF, PIK3CA, PTEN, or AKT1 | Present; absent |
| TP53 mutation analysis | Gene-specific analysis | Pathogenic or likely pathogenic TP53 sequence variant retained after filtering and annotation | Present; absent |
| Chromosome 12p analysis | Copy-number analysis | Chromosome 12p gain defined by coordinated copy-number increase across ≥3 contiguous 12p target regions | Present; absent |
| KRAS amplification analysis | Gene-level copy-number analysis | KRAS amplification defined as estimated copy number ≥5.0 | Present; absent |
| Exploratory genomic grouping | Adverse genomic signature | One qualifying feature for each TP53 pathogenic or likely pathogenic mutation, chromosome 12p gain, KRAS amplification, and each additional pathogenic or likely pathogenic oncogenic alteration; signature-positive at ≥2 total qualifying features | Signature-positive; signature-negative |
| Primary endpoint | Event-free survival | Time from first pathological diagnosis to relapse, progression, second-line treatment for persistent disease, death from any cause, or last follow-up | Months |
| Secondary endpoint | Overall survival | Time from first pathological diagnosis to death from any cause or last follow-up | Months |
| Censoring rule for EFS | Event-free censoring | Patients without relapse, progression, second-line treatment, or death were censored at last confirmed follow-up | Censored observation |
| Censoring rule for OS | Overall survival censoring | Patients alive at last confirmed follow-up were censored | Censored observation |
| Follow-up duration | Median follow-up | Calculated using the reverse Kaplan–Meier method | Median months with IQR or range |
| Survival estimation | EFS and OS estimation | Kaplan–Meier method | Survival estimate with 95% CI |
| Survival comparison | Group comparison | Log-rank test | P value |
| Univariable Cox regression | Candidate clinicopathological predictors | Age group, primary site, tumor stage, histological subtype, tumor size category, AFP elevation, β-hCG elevation, residual disease, metastatic disease, and chemotherapy exposure | HR, 95% CI, P value |
| Univariable Cox regression | Candidate genomic predictors | Recurrent genomic alterations, RTK/RAS/PI3K pathway alteration, TP53 mutation, chromosome 12p gain, KRAS amplification, and adverse genomic signature | HR, 95% CI, P value |
| Main multivariable Cox model | Adjusted model | Tumor stage and adverse genomic signature | Adjusted HR, 95% CI, P value |
| Missing data | Primary handling method | Complete-case analysis | Number of cases included in each analysis |
| High missingness rule | Variable exclusion threshold | Variables with >20% missing values excluded from regression modeling | Descriptive reporting only |
| Imputation | Missing-data imputation | No statistical imputation performed | Not applicable |
| Dataset lock | Analysis dataset | Final cleaned clinical and genomic dataset used for all statistical analyses | Locked before survival modeling |

SD = standard deviation; IQR = interquartile range; EFS = event-free survival; OS = overall survival; HR = hazard ratio; CI = confidence interval.

Supplementary Figure S1. Hypothesis-generating genomic–microenvironment framework. Arrows denote proposed relationships rather than measured effects. This conceptual figure presents no quantitative immunohistochemical data. EFS = event-free survival; IHC = immunohistochemistry.
